# The effects of Brazilian green propolis that contains flavonols against mutant copper-zinc superoxide dismutase-mediated toxicity

**DOI:** 10.1038/s41598-017-03115-y

**Published:** 2017-06-06

**Authors:** Tomoyuki Ueda, Masatoshi Inden, Katsuhiro Shirai, Shin-ichiro Sekine, Yuji Masaki, Hisaka Kurita, Kenji Ichihara, Takashi Inuzuka, Isao Hozumi

**Affiliations:** 1 Lab. Medical Therapeutics and Molecular Therapeutics, Gifu Pharmaceutical Univ, Gifu, Japan; 2 Nagaragawa Research Center, Api Company Limited, Gifu, Japan; 3 Department of Neurology and Geriatrics, Gifu University Graduate School of Medicine, Gifu, Japan

## Abstract

Amyotrophic lateral sclerosis (ALS) is a fatal neurodegenerative disease characterized by the selective and progressive loss of motor neurons. The purpose of this study was to clarify effects of brazilian green propolis and the active ingredient against ALS-associated mutant copper-zinc superoxide dismutase (SOD1)-mediated toxicity. Ethanol extract of brazilian green propolis (EBGP) protected N2a cells against mutant SOD1-induced neurotoxicity and reduced aggregated mutant SOD1 by induction of autophagy. Kaempferide and kaempferol, the active ingredients of EBGP, also inhibited mutant SOD1-induced cell death and reduced the intracellular mutant SOD1 aggregates. Both kaempferide and kaempferol significantly suppressed mutant SOD1-induced superoxide in mitochondria. Western blot analysis showed that kaempferol potentially induced autophagy *via* the AMP-activated protein kinase (AMPK) - the mammalian target of rapamycin (mTOR) pathway. These results suggest that EBGP containing the active ingredient against mutant SOD1-mediated toxicity is a promising medicine or health food for prevention and treatment of ALS.

## Introduction

Amyotrophic lateral sclerosis (ALS) is characterized by the degeneration of motor neurons and by the formation of intracellular protein aggregations that form in motor neurons. While 95% of ALS is sporadic, 5% is inherited. Among the inherited ALS (also known familial ALS (FALS)), the mutations in copper-zinc superoxide dismutase (SOD1) are the major autosomal dominant inherited cause for ALS^1^. Mutant SOD1 proteins form insoluble aggregations with components of the ubiquitin proteasome system (UPS) and autophagy pathway in motor neurons^2, 3^. The relationship between motor neuron death and mutant SOD1 aggregations remain elusive. The excess of mutant SOD1 aggregations decreased the elimination capacity of the UPS^3–5^. The impairment of the UPS by the excess aggregations induced endoplasmic reticulum (ER) stress, mitochondrial dysfunction, and oxidative stress^3, 6^. On the other hand, several evidences have suggested that autophagy activation alleviates mutant SOD1-linked toxic insults^7, 8^. In addition, Mahogunin ring finger-1 (MGRN1) E3 ubiqutin ligase, which catalyzes mono-ubiquitination to the substrate, contributes to the clearance of mutant SOD1 aggregations likely *via* autophagic pathway^8^.

Propolis is made from a sticky substance that honeybees produce by mixing their own waxes with resinous sap obtained from the bark and leaf-buds of certain trees. The color of propolis can be green, red, brown, or almost black depending on the plants from which the resinous substance is collected. The properties and constituents of propolis also differ with its geographical origin. Propolis presents numerous biological and pharmacological properties, such as anti-bacterial, anti-inflammatory, and anti-oxidative activity^9–13^. The previous our study also showed that propolis promoted the advantage of the conditioned medium of dental pulp cells *via* producing neurotrophic factors^13^. However, the effect of propolis and the active components against ALS-associated mutant SOD1-mediated toxicity is not well known.

In the present study, to examine whether propolis and the active components have neuroprotective effect against mutant SOD1-induced neurotoxicity in a cellular model, we used the ethanol extract of Brazilian green propolis (EBGP). In Japan, Brazilian green propolis has recently come to be used as a health food, which is of sufficiently good quality to provide experimental reproducibility. The ethanol extract of propolis is the major form used in health food^14^. We also further investigate whether autophagy is involved in the neuroprotection of kaempferol and kaempferide against mutant SOD1-related neurotoxicity via the AMP-activated protein kinase (AMPK) - the mammalian target of rapamycin (mTOR) pathway.

## Results

### EBGP reduced these intracellular aggregates of SOD1^G85R^ and prevented SOD1^G85R^-induced neurotoxicity

**Figure 1:**
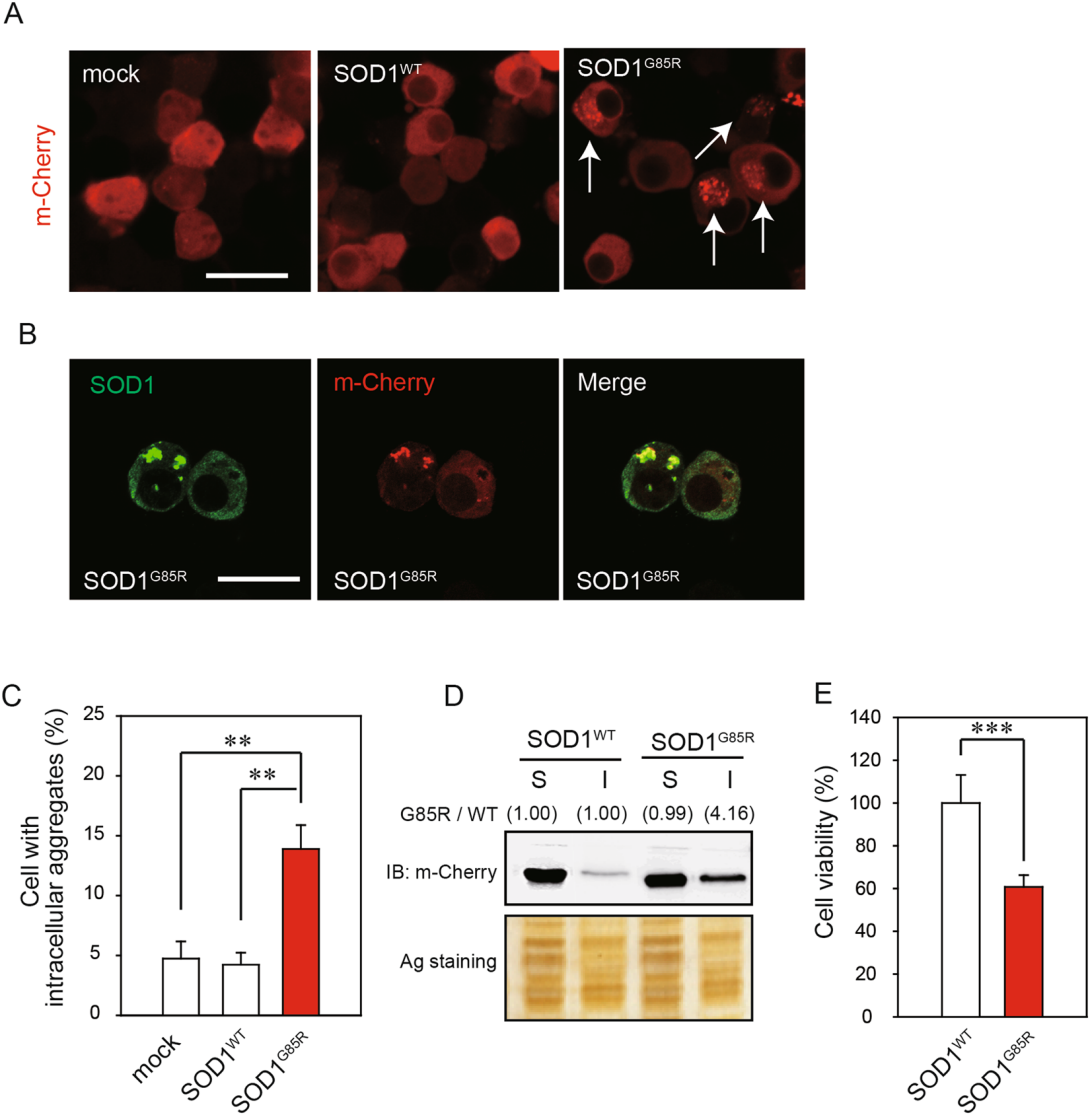
SOD1^G85R^ caused neurotoxicity *via* the formation of insoluble aggregates. (**A**) Representative fluorescent microscopy images of N2a cells expressing mCherry-SOD1^WT^ or mCherry-SOD1^G85R^. The arrowheads indicate the intracellular SOD1 aggregates. (**B**) Colocalization of SOD1 and mCherry in the N2a cells. (**C**) Quantified data of intracellular SOD1 aggregates are expressed as mean ± S.E.M from three independent experiments. In each experiment, at least 200 cells were counted. (**D**) Immunoblot analysis of SOD1 aggregates. N2a cells expressing SOD1 constructs were lysed with 1% TritonX-100 (Triton soluble fraction; S). Triton-insoluble fraction (I) were resuspended with 2% SDS and then analyzed with immunoblotting by anti-mCherry antibody. (**E**) N2a cells expressing mCherry-SOD1^WT^ or mCherry-SOD1^G85R^ were incubated for 48 h in the differentiation medium. The cell viability was measured by MTT assay. **p < 0.01, ***p < 0.001. Scale bar: 50 µm.

Currently, more than 150 types of pathogenic mutations in SOD1 gene have been identified in ALS patients^15^. Among these mutations, the pathogenic SOD1^G85R^ mutation has been frequently studied^16^. The transgenic mice having transgene of SOD1^G85R^ mutation show rapidly progressive motor neuron degeneration. The aggregate formation was confirmed in SOD1^G85R^-transfected N2a cells based on previous study^8, 17–19^. As shown in Fig. 1A, the mCherry-fused SOD1^G85R^ (thereafter SOD1^G85R^) formed intracellular aggregates in approximately 15% of total transfected N2a cells, whereas mCherry-fused SOD1^WT^ (thereafter SOD1^WT^) was distributed evenly in the cytoplasm (Fig. 1A,C). Immunostaining was performed to confirm that the signal of mCherry was SOD1. As shown in Fig. 1B, the signal of mCherry colocalized with the signal of SOD1. Similarly to previous studies^17, 18^, western blot analysis showed that triton X-100-insoluble SOD1^G85R^ was increased (Fig. 1D). In order to examine SOD1^G85R^-mediated toxicity to differentiated N2a cells, we performed MTT assay. Although SOD1^WT^ did not affect cell survival rate, SOD1^G85R^ induced cell death (Fig. 1E). These results suggest that SOD1^G85R^ caused cell death *via* the formation of insoluble aggregates.

**Figure 2:**
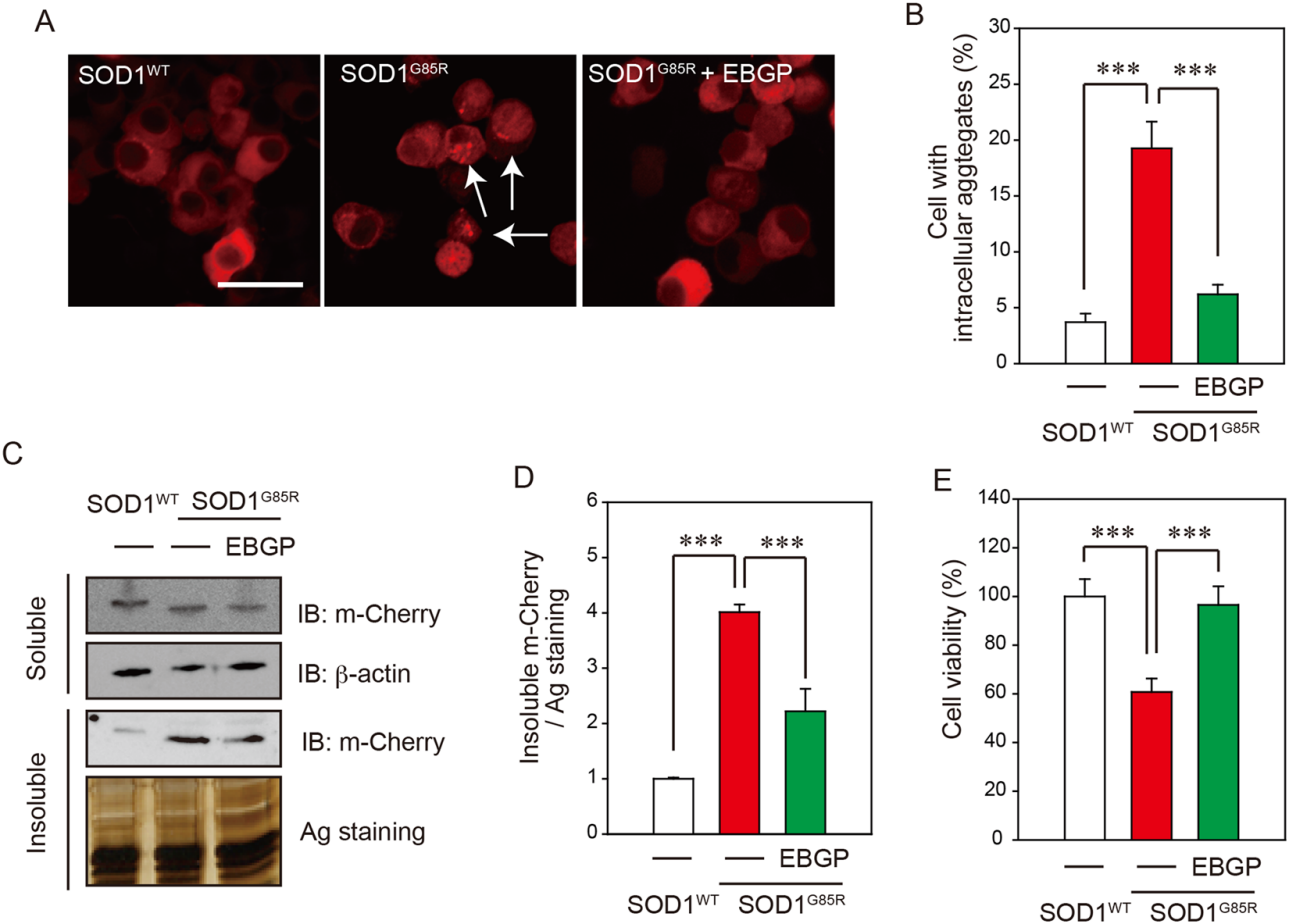
EBGP protected SOD1^G85R^-induced neurotoxicity *via* the reduction of the aggregates of SOD1^G85R^. (**A**) Representative fluorescent microscopy images of N2a cells expressing mCherry-SOD1^G85R^ incubated for 24 h with 20 ng/mL EBGP. The arrowheads indicate the intracellular SOD1 aggregates. (**B**) Quantified data of intracellular SOD1 aggregates are expressed as mean ± S.E.M from three independent experiments. In each experiment, at least 200 cells were counted. (**C**) Reduction of Triton-insoluble mutant SOD1^G85R^ by EBGP. After treatment of 20 ng/mL EBGP, N2a cells expressing SOD1^G85R^ were lysed with 1% TritonX-100. Triton-insoluble fraction was resuspended with 2% SDS and analyzed with immunoblotting. (**D**) The density of Triton-insoluble mutant SOD1^G85R^ is given as mean ± S.E.M from three independent experiment, based on the density of Triton-insoluble mutant SOD1^WT^. (**E**) N2a cells expressing mCherry-SOD1^G85R^ were treated with 20 ng/mL EBGP. The cell viability was measured by MTT assay. ***p < 0.001. Scale bar: 50 µm.

Next, to investigate the effect of EBGP against SOD1^G85R^ aggregates, we evaluated the number of intracellular SOD1^G85R^ aggregates. EBGP significantly reduced these intracellular aggregates of SOD1^G85R^ to approximately 30% of untreated cells (Fig. 2A,B). Western blot analysis also showed that triton X-100-insoluble SOD1^G85R^ was significantly decreased by treatment of EBGP, although triton X-100-soluble SOD1^G85R^ did not change (Fig. 2C,D). In addition, EBGP prevented SOD1^G85R^-induced neurotoxicity (Fig. 2E). There results suggest that the active components of EBGP have significant effects for the neuroprotection against SOD1^G85R^-induced neurotoxicity.

### The active component of EBGP, kaempferol and kaempferide, are involved in neuroprotection against mutant SOD1-related toxicity

**Figure 3:**
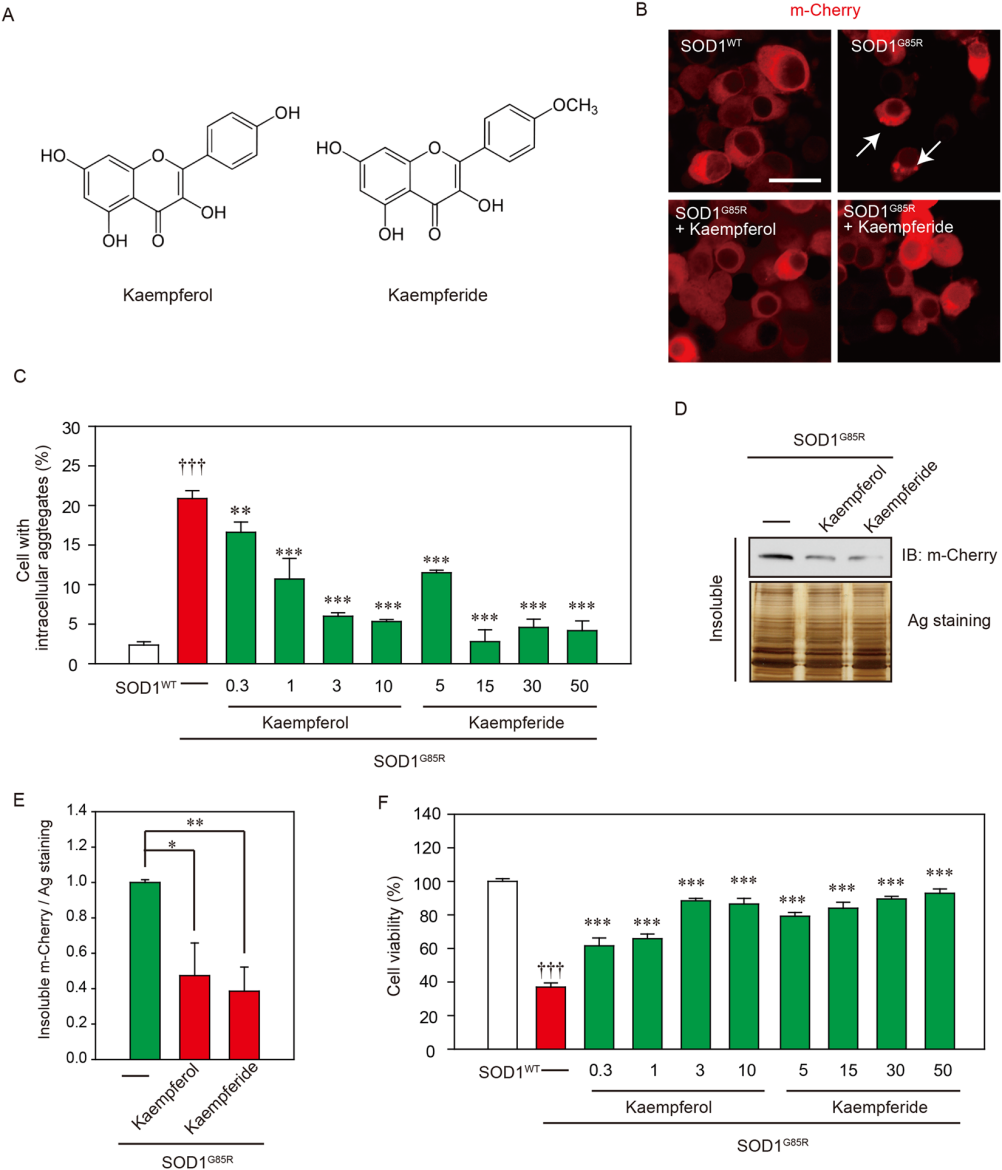
Kaempferol and kaempferide prevented SOD1^G85R^-related neurotoxicity. (**A**) Chemical structures of kaempferol and kaempferide. (**B**) Representative fluorescent microscopy images of N2a cells expressing mCherry-SOD1^G85R^ incubated for 24 h with 3 µM kaempferol or 15 µM kaempferide. (**C**) Quantified data of intracellular SOD1 aggregates in N2a cells expressing mCherry-SOD1^G85R^ incubated for 24 h with kaempferol (from 0.3 µM to 10 µM) or kaempferide (from 5 µM to 50 µM) are expressed as mean ± S.E.M from three independent experiments. In each experiment, at least 200 cells were counted. (**D**) Reduction of Triton-insoluble mutant SOD1^G85R^ by kaempferol and kaempferide. After treatment of 3 µM kaempferol or 15 µM kaempferide, Triton-insoluble fraction of N2a cells expressing SOD1^G85R^ was resuspended with 2% SDS and analyzed with immunoblotting. (**E**) The band density of Triton-insoluble mutant SOD1^G85R^ group is given as mean ± S.E.M from three independent experiments, based on the band density of Triton-insoluble non-treated mutant SOD1^G85R^ group. (**F**) N2a cells expressing mCherry-SOD1^G85R^ were treated with kaempferol (from 0.3 µM to 10 µM) or kaempferide (from 5 µM to 50 µM). The cell viability was measured by MTT assay. Data is expressed as mean ± S.E.M from three independent experiments, based on SOD1^WT^ group. *p < 0.05; **p < 0.01; ***p < 0.001. Scale bar: 20 µm.

Previous study reported that HPLC analysis shows the major chemical constituents of the EBGP are phenolic acids/flavonoids, such as artepillin C, baccharin, drupanin, *p*-coumaric, Betuletol, kaempferide and kaempferol^20^. Among these components, we chose two flavonoids, kaempferol and kaempferide (Fig. 3A). We examined effects of kaempferol and kaempferide against SOD1^G85R^ aggregates. As shown in representative photomicrographs, kaempferol and kaempferide remarkably reduced the intracellular aggregates of SOD1^G85R^ (Fig. 3B). We further examined the effect of kaempferol and kaempferide on SOD1 aggregation by using a series of concentrations based on previous studies (Fig. 3C)^21, 22^. As results, kaempferol (from 0.3 µM to 10 µM) and kaempferide (from 5 µM to 50 µM) significantly decreased the aggregates. Western blot analysis also showed that triton X-100-insoluble SOD1^G85R^ was significantly decreased by treatment of kaempferol and kaempferide (Fig. 3D,E). Moreover, we showed that kaempferol and kaempferide prevented SOD1^G85R^-induced neurotoxicity (Fig. 3F and Supplementary Fig. S1). These results suggest that kaempferol and kaempferide are involved in neuroprotection against mutant SOD1-related toxicity.

### The anti-oxidant activity is involved in the neuroprotection of kaempferol and kaempferide against mutant SOD1-related toxicity

**Figure 4:**
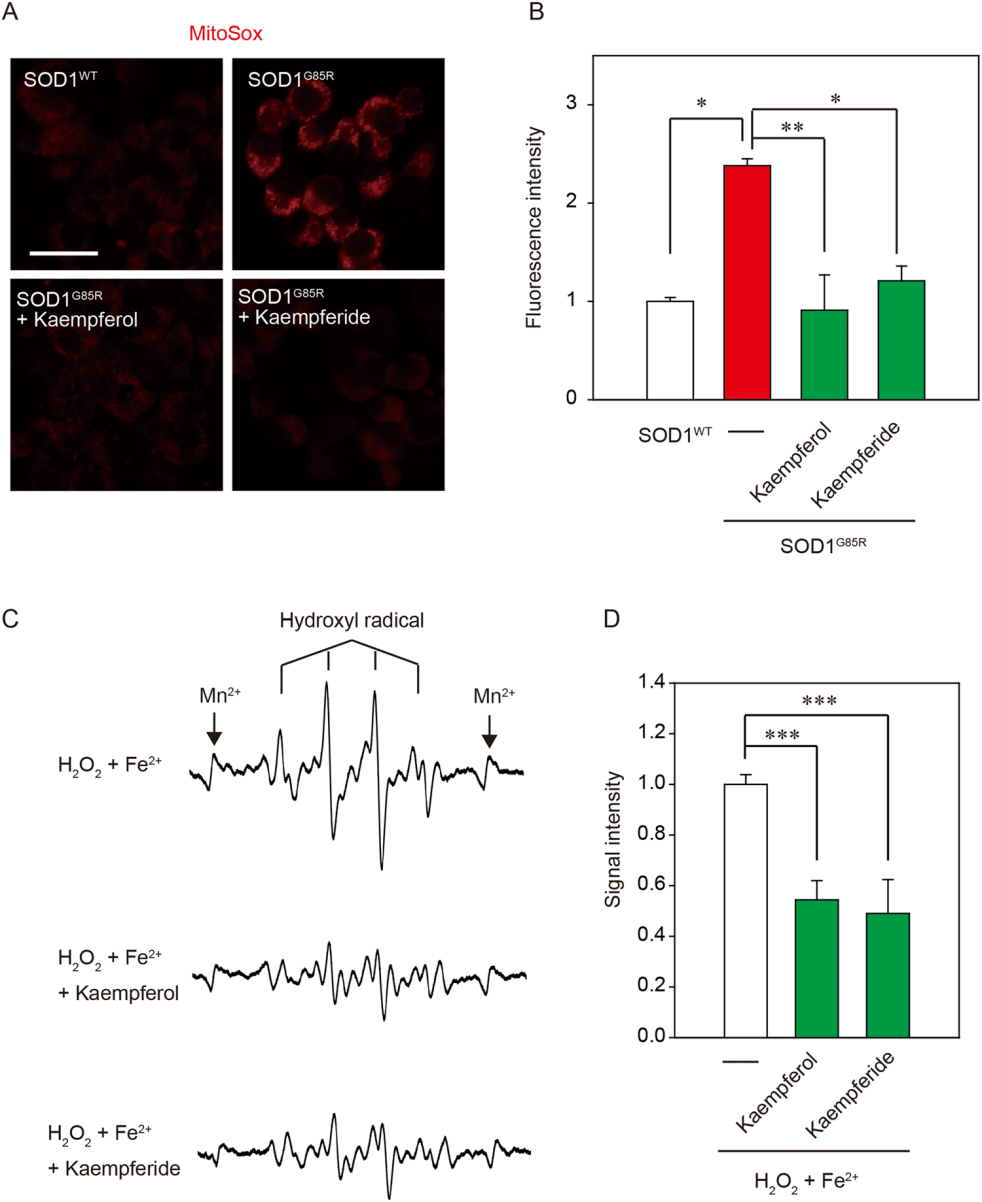
Both kaempferol and kaempferide suppressed SOD1^G85R^-induced superoxide in mitochondria. (**A**) N2a cells expressing mCherry-SOD1^G85R^ were treated with 3 µM kaempferol or 15 µM kaempferide for 24 h. Subsequently, MitoSOX® Red was added to the cell culture to a final concentration of 5 μM to the cells and incubated for 30 min at 37 °C. (**B**) The relative fluorescence intensity was quantified by computerized image analysis with ImageJ. Data are expressed as mean ± S.E.M from three independent experiment, based on the fluorescence intensity of SOD1^WT^. (**C**) Traces show typical spectra of DMPO-OH spin adducts generated from H_2_O_2_ plus Fe^2+^ in the absence (control) or presence of kaempferol and kaempferide. (**D**) The amount of •OH was semi-quantitatively measured as the formation of DMPO-OH spin adducts by ESR spectrometry. Data is expressed as the mean ± S.E.M of three determinations, based on control. *p < 0.05; **p < 0.01. ***p < 0.001. Scale bar: 50 µm.

Previous study showed that flavonoid exerts an anti-oxidant activity^23^. Because oxidative stress is well recognized as an important pathogenic event in ALS, the effects of kaempferol and kaempferide against SOD1^G85R^-induced ROS were examined using MitoSOX Red, a mitochondrial-targeted superoxide sensitive fluorogenic probe^24^. The increase in MitoSOX Red fluorescence in N2a cells transfected GFP-SOD1^G85R^ was significantly attenuated by kaempferol and kaempferide (Fig. 4A,B). To clarify the direct scavenging effect of kaempferol and kaempferide against ROS such as hydroxyl radical (•OH), we performed electron spin resonance (ESR) analysis (Fig. 4C,D). As result, both kaempferol and kaempferide presented potent scavenging activity against •OH. These results demonstrate that the anti-oxidant activity is involved in the neuroprotection of both kaempferol and kaempferide against mutant SOD1-related neurotoxicity.

### Kaempferol, but not kaempferide, reduced intracellular aggregate *via* the activation of autophagy

**Figure 5:**
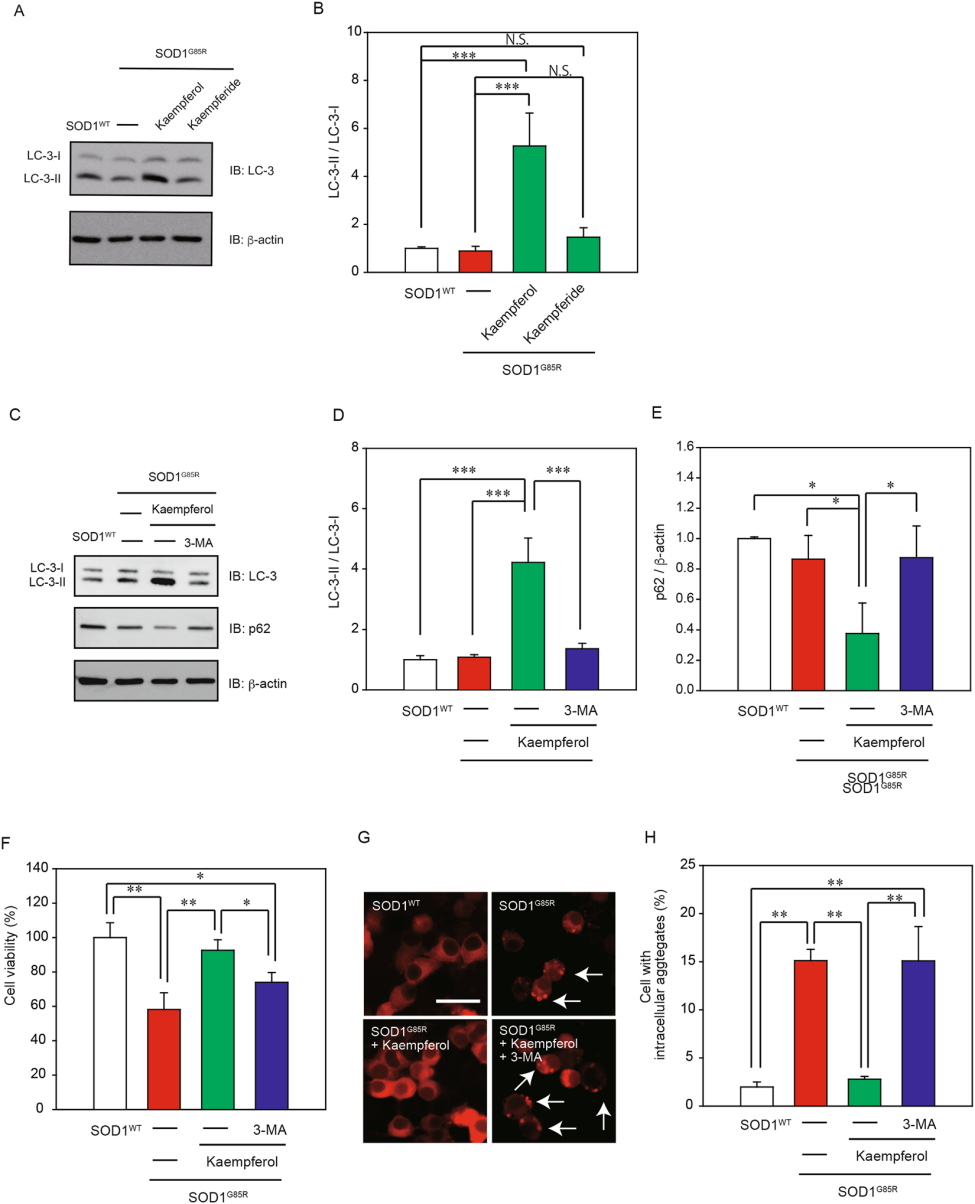
Kaempferol, but not kaempferide, reduced the intracellular aggregates *via* the activation of autophagy. (**A**) Induction of LC-3-II by kaempferol, but not kaempferide. N2a cells were transfected with SOD1^G85R^, and then incubated with 3 µM kaempferol or 15 µM kaempferide for 24 h. The lysates were analyzed by immunoblotting with anti-LC-3 and anti-β-actin antibodies. (**B**) Relative levels of LC-3-II/LC-3-I normalized by the expression of β-actin were quantified, based on the band density of SOD1^WT^. (**C**–**E**) Immunoblotting analysis of autophagy. N2a cells expressing SOD1^G85R^ were treated with kaempferol for 24 h with and without 3-MA. The lysates were analyzed by immunoblotting with antibodies for LC-3, p62 and β-actin. Relative levels of LC-3-II/LC-3-I and p62 normalized by the expression of β-actin were quantified, based on the band density of SOD1^WT^. (**F**) Representative fluorescent microscopy images of N2a cells expressing mCherry-SOD1^G85R^ incubated for 24 h with 3 µM kaempferol with and without 3-MA. (**G**) Quantified data of intracellular SOD1 aggregates are expressed as mean ± S.E.M from three independent experiments. In each experiment, at least 200 cells were counted. (**H**) N2a cells expressing SOD1^G85R^ was incubated for 24 h with 3 µM kaempferol with and without 3-MA. Cell viability was determined. Data is expressed as mean ± S.E.M from three independent experiments. *p < 0.05; **p < 0.01. ***p < 0.001.

Previous study reported that flavonol induced autophagy^21^. To further investigate whether autophagy is involved in the neuroprotection of kaempferol and kaempferide against mutant SOD1-related neurotoxicity, western blot analysis was performed using LC3 antibody, a selective marker of autophagy. Western blot analysis showed that kaempferol, but not kaempferide, enhanced the formation of LC3-II (Fig. 5A,B). Western blot analysis was also performed using p62 antibody, another selective marker of autophagy. As shown in Fig. 5C–E, the expression of LC-3-II/LC-3-I was increased while that of p62 was decreased after treatment with kaempferol. In addition, the change of LC-3-II/LC-3-I and p62 levels was improved by 3-methyladenine (3-MA), an inhibitor of autophagy, indicating the autophagy is activated by kaempferol. To confirm the relationship between neuroprotective effect of kaempferol against mutant SOD1-mediated toxicity and induction of autophagy, the MTT assay was performed with 3-MA. The protective effect of kaempferol was significantly abolished by 3-MA treatment (Fig. 5F). In addition, immunofluorescence staining showed that the reduction of intracellular aggregates of SOD1^G85R^ caused by kaempferol was significantly abolished by 3-MA treatment (Fig. 5G,H). These results suggest that kaempferol, but not kaempferide, reduced intracellular aggregate *via* the activation of autophagy and then inhibited mutant SOD1-induced neurotoxicity.

### Kaempferol increases AMPK phosphorylation

**Figure 6:**
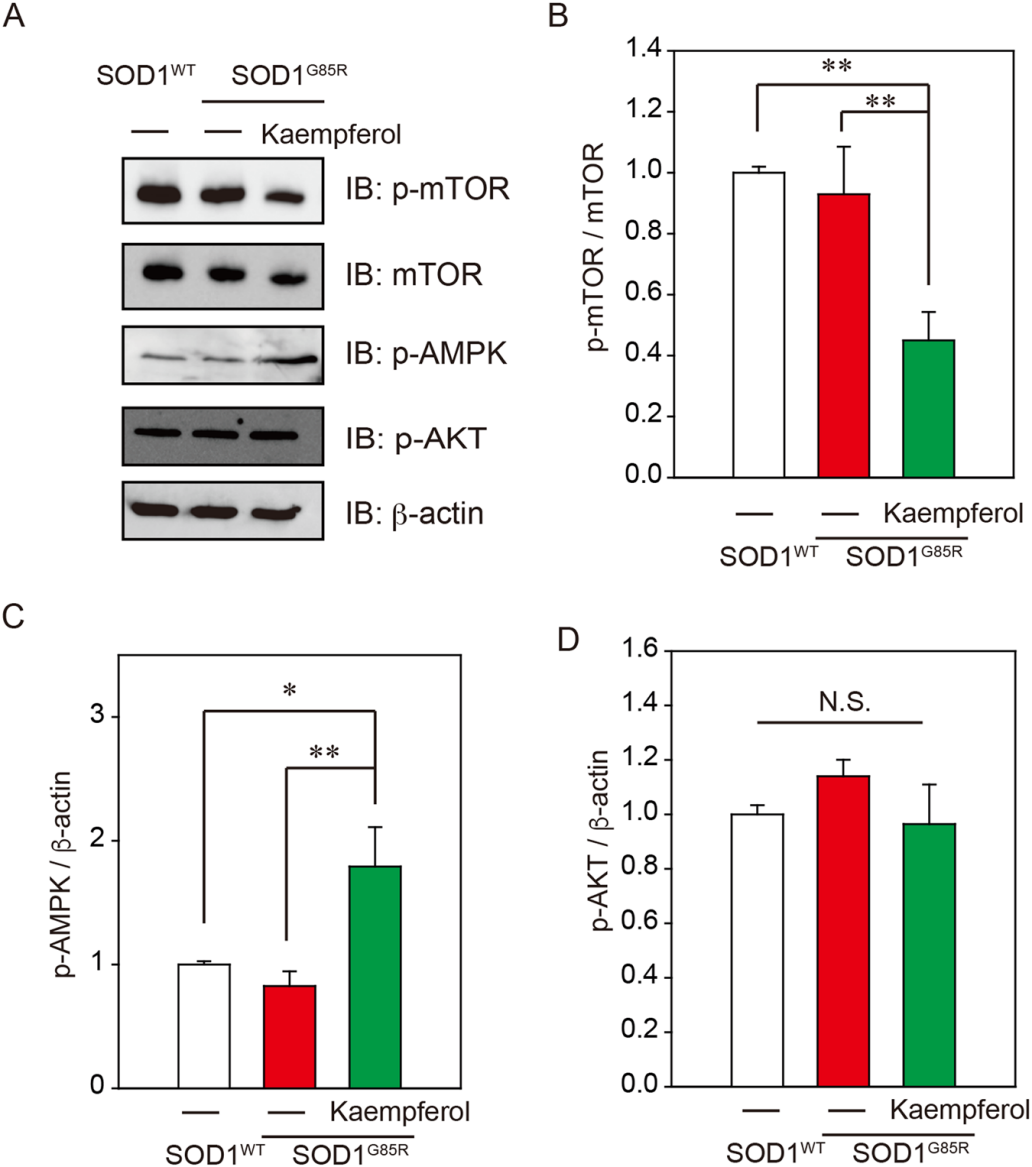
Kaempferol increases AMPK phosphorylation. (**A**) Immunoblotting analysis of autophagy regulators. N2a cells expressing SOD1^G85R^ were treated with 3 µM kaempferol for 24 h. The lysates were analyzed by immunoblotting with antibodies for phosphorylated mTOR (p-mTOR), mTOR, phosphorylated AMPK (p-AMPK), phosphorylated AKT (p-AKT), β-actin. (**B**) Relative levels of p-mTOR normalized by the expression of mTOR were quantified, based on the density of SOD1^WT^. (**C**,**D**) Relative levels of p-AMPK and p-AKT normalized by the expression of β-actin were quantified, based on the band density of SOD1^WT^. *p < 0.05. N.S.; not significant.

Previous study reported that flavonol inhibited the mammalian target of rapamycin (mTOR) complex, which suppresses autophagy^22^. However, the mechanisms through which kaempferol inhibits mTOR are still unclear. To identify the signal transduction pathway mediated by kaempferol, we investigated AMPK and AKT pathways. AMPK inactivates mTOR, and AKT induced mTOR activity. Kaempferol significantly inhibited mTOR phosphorylation (Fig. 6A,B) and significantly increased AMPK phosphorylation (Fig. 6A,C). On the other hand, AKT phosphorylation was not affected by kaempferol (Fig. 6A,D). These results suggest that kaempferol potentially induces autophagy *via* the AMPK-mTOR pathway.

## Discussion

Many studies have been conducted to elucidate the health benefits and the mechanisms of EBGP, including studies on its effects on bacteria^9^, viruses^10^, inflammation^11^, and cancer^12^. However, little has been reported on effects of EBGP for neurodegenerative disease such as ALS. Dominant mutations in SOD1 are frequent cause of inherited ALS^1^. Misfolded and aggregated SOD1 is present in both sporadic and familial ALS, although its pathobiology remains unclear^25, 26^. A reduction of misfolded and aggregated SOD1 might be one of potential therapeutic approaches for ALS^17^. Therefore, in the present study, we examine whether EBGP have neuroprotective effect against SOD1^G85R^ -induced neurotoxicity in a cellular model for ALS. EBGP prevented SOD1^G85R^-induced neurotoxicity. Brazilian green propolis has been reported to contain many biological active compounds^27^. There results suggest that the active components of EBGP play an important for the neuroprotection against SOD1^G85R^-induced neurotoxicity.

Propolis is collected by honeybees from various plants. There are different types of propolis according to its botanical origin and geographical region. Brazilian green propolis has been reported to contain many biological active compounds, including cinnamic acid derivatives (e.g., artepillin C, baccharin, drupanin, and *p*-coumaric acid) and flavonoids (e.g., kaempferol, and kaempferide)^20, 27^. Flavonoids are considered as an important part of the anti-oxidants in food. Kaempferol and kaempferide are known to have an anti-oxidative activity^23^. In addition, previous study reported that kaempferol induced autophagy, although the mechanism is unclear^21, 22^. Therefore, because oxidative stress and autophagy are well recognized as an important pathogenic event in ALS, we chose two flavonoids, kaempferol and kaempferide among these components in Brazilian green propolis, and examined effects of these flavonoids against mutant SOD1-related toxicity.

In the present study, separation of protein fraction by using triton X-100 was performed according to published protocols^17, 18^. We also performed immunocytochemical assay based on previous studies^8, 17–19^. In addition, previous studies have shown that progressive accumulation of mutant SOD1, which is correlated with a decrease of its solubility in triton X-100, is observed in the spinal cord of SOD1 tg mice, and evident aggregates of SOD1 are found at a late stage of the disease in SOD1 tg mice^28, 29^. Considering the evidence of these previous studies, we selected the triton X-100 as a detergent in the present study.

Numerous studies have identified significant mitochondrial abnormalities in mutant SOD1-associated ALS^24, 30, 31^. In addition, mutant SOD1 forms insoluble aggregates in mitochondria at the surface of outer membrane, raising the prospect of a direct cause-effect mechanism by which mutant SOD1 impact mitochondrial function^32, 33^. Taken together, these studies provide reasonable evidence for the involvement of mitochondrial-produced oxidants and mitochondrial oxidative damages in mutant SOD1-related ALS. The cause and effect of increased mitochondrial produced ROS in mutant SOD1-induced mitochondrial dysfunction requires further study. However, the present results herein showing that the active components such as kaempferol and kaempferide of Brazilian green propolis inhibited mutant SOD1-induced superoxide suggest that Brazilian green propolis and the active components might be utilized to promote the therapeutic strategy for ALS.

The propensity of a flavonoid to inhibit free-radical mediated events is governed of the chemical structure. Previous study showed that flavonoids with 3-hydroxy (OH) group play a positive role in anti-oxidative activity^23^. Kaempferol and kaempferide are contained in 3-OH group. Therefore, the chemical structure of flavonoid having the 3-OH group may contribute to the anti-oxidative activity against mutant SOD1-induced ROS. On the other hand, kaempferol, but not kaempferide, induces autophagy *via* the AMPK-mTOR pathway in the present study. The difference in the chemical structure of flavonoid is only functional group in the B ring between kaempferol and kaempferide. Kaempferol and kaempferide frame with the B ring 4′-OH group and 4′-methoxy group, respectively (Fig. 3). There are differences in beneficial actions such as anti-oxidative activity according to the structure of the B ring of flavonoids^23^. Therefore, kaempferol having 4′-OH group in the B ring may be related to the autophagy, although further studies are needed to elucidate the relation between the chemical structure of flavonoid and the autophagy. On the other hand, to the best of our knowledge, there is no report that flavonoids including kaempferol and kaempferide bind to mutant SOD-1 like a chemical chaperone. In the present study, it is unknown whether kaempferol and kaempferide directly bind to mutant SOD1. It remains in future study. Further research is required to provide more detail of interaction experiments using recombinant mutant SOD1.

In the present study, kaempferol increased AMPK phosphorylation to inhibit mTOR phosphorylation, and subsequent induced autophagy. Previous study demonstrated that AMPK was inactivated both in the *in vitro* and *in vivo* models of SOD1-associated ALS and that the reduced activation of AMPK by mutant SOD1 was recovered by cystain C treatment^17^. In addition, resveratrol protective effects were associated with increased expression and activation of AMPK in spinal cords of SOD1^G93A^ mice^32^. Moreover, riluzole, an approved ALS drug, leads to AMPK phosphorylation^33^. However, several previous studies demonstrated that the reduced activated of AMPK improved the mutant SOD1-induced motor neuron death *in vitro* and the motor activity of neurons *in vivo* ^34^. These results suggest that an imbalance in energy metabolism should be considered an important factor in both progression and potential treatment of ALS and that AMPK play a key role in the disease. Therefore, kaempferol as well as EBGP containing the active ingredient against mutant SOD1-mediated toxicity will be an efficient source of new treatments and prevention for ALS.

## Methods

### Plasmid, cell culture, and transfection

Wild-type human SOD1 cDNA was purchased from TransOMIC Technologies, and subcloned into pmCherry-N1 (Clontech Laboratories Inc., CA, USA) between the HindIII/BamHI sites (pmCherry-SOD1^WT^). The mutant SOD1 gene was generated by Quick Change site-directed mutagenesis (Stratagene, La Jolla, CA, USA) according to the manufacturer’s protocol (pmCherry-SOD1^G85R^). The primer pairs were as follows: 5′-GTTAAGCTTATGGCGACGAAGGCCGTGTGC-3′ and 5′-GCAGGATCCGGTTGGGCGATCCCAATTACACC-3′ for pmCherry-SOD1^WT^, 5′-GACTTGCGCAATGTGACTGCTGACAAA-3′ and 5′-CACATTGCGCAAGTCTCCAACATGCCT-3′ for pmCherry-SOD1^G85R^.

N2a cells were maintained in Dulbecco’s modified Eagle medium (DMEM, Wako Pure Chemical Industries, Ltd., Osaka Japan) containing 10% (v/v) fetal bovine serum (FBS; Thermo Fisher Inc., Waltham, MA, USA) under a humidified atmosphere of 5% CO_2_ at 37 °C. The cells were passaged by trypsinization every 3–4 days. Transient expression of each plasmid in N2a cells was accomplished with Lipofectamine 2000 according to the manufacturer’s protocol (Thermo Fisher Scientific Inc., Waltham, MA, USA).

### Aggregation rate analysis

After 24 h of transfection of each vector to N2a cells, the cells were treated with or without EBGP (a gift from the Api Co., Ltd., Gifu, Japan), kaempferol (Wako Pure Chemical Industries Ltd., Osaka, Japan), or kaempferide (INDOFINE Chemical Company) in the presence or absence of 3-methyladenine (3-MA) for 24 h. Subsequently, the cells were washed with PBS for 5 min twice and fixed with 4% paraformaldehyde for 15 min. Fluorescent microscopy images were acquired using a confocal fluorescence microscope (LSM700, Carl Zeiss, Jena, Germany) and the images were analyzed using ZEN software (Carl Zeiss). Aggregates were defined as bright non-homogeneities with the cytoplasm. To quantify aggregate formation, the intensity of the even and diffuse fluorescence was set as threshold and the number of cells in each field showing fluorescence intensities over the threshold was counted using imaging software. Following the threshold analysis, the presence of aggregates was confirmed by eye in each field, based on previous studies^8, 17–19^.

### Western blot analysis

After 24 h of transfection to N2a cells each vector, the cells were treated with or without EBGP, kaempferol, or kaempferide. The cells were lysed with TNE lysis buffer (50 mM Tris-HCl (pH. 7.4), 150 mM NaCl, 1 mM ethylenediamineteraacetic acid, protease inhibitor cocktail) containing 1% Triton-X and then were centrifuged at 15,000 × g for 5 min at 4 °C. The supernatant of the lysate was defined as Triton soluble fraction. After centrifuge, the remaining deposition was resuspended with TNE lysis buffer containing 2% sodium dodecyl sulfate (SDS) (defined as Triton-insoluble fraction). Protein concentrations were measured using a BCA protein assay kit (Thermo Fisher Scientific Inc.) with bovine serum albumin (BSA) as a standard. Lysates were mixed with sample buffer containing 10% 2-mercaptoethanol, and subjected to 10% SDS-polyacrylamide gel electrophoresis (SDS-PAGE). The 20 µg of protein was applied to SDS-PAGE in order to separate proteins as certain molecular weight. SDS-PAGE was performed under constant voltage at 200 V at room temperature for 40 min. The separated proteins in poly acrylamide gel were transferred to PVDF membrane in transfer buffer (0.3% Tris, 1.44% glycine, 20% methanol) under constant voltage at 100 V at 4 °C for 90 min. The membranes were incubated with 5% BSA (Wako) for 60 min, and then with following primary antibodies for overnight: mouse monoclonal antibodies against mCherry (1:1000, Clontech), β-actin (1:2000, Santa Cruz Biotechnology, Dallas, TX USA),; rabbit polyoclonal antibodies: LC-3 (1:1000, Cell Signaling Technology, Danvers, MA USA), p-AKT (1:1000, Cell Signaling Technology), AKT (1:1000, Cell Signaling Technology), p-AMPK (1:1000, Cell Signaling Technology), p-mTOR (1:1000, Cell Signaling Technology), mTOR(1:1000, Cell Signaling Technology), p62 (1:1000, Cell Signaling Technology). After the primary antibody reaction, membrane was incubated in the second antibody (goat anti-rabbit antibody conjugated with HRP (1:2500, Santa Cruz Biotechnology), goat anti-mouse HRP antibody conjugated with HRP (1:2500, Santa Cruz Biotechnology)). The membrane was incubated in ECL prime (GE Healthcare, Buckinghamshire, UK) to generate the chemiluminescence from HRP antibodies. The chemiluminescence was detected by using LAS3000 mini (Fuji film, Tokyo, Japan). The band density was measured by ImageJ software (NIH, New York, NY, USA).

### Neurotoxicity assays

N2a cells were seeded at 2.0 × 10^5^ cells/ml in 96-well plates in DMEM containing 10% FBS. After transfection of each plasmid, the cells were differentiation for 48 h in low glucose (1.0 g/l glucose) DMEM supplemented with 2% FBS and 2 mM N,N-dibutyladenosine 3′,5′-phosphoric acid (dbcAMP; Nacalai tesque, Kyoto, Japan) with or without EBGP, kaempherol, or kaempheride in the presence or absence of 3-MA. The number of live cells was estimated by Cell Counting Kit-8, following the manufacturer’s instructions (Wako Pure Chemical Industries Ltd.). Briefly, reagent was added into wells and the plate was incubated at 37 °C for 4 h. The optical density of formazan was detected at 450 nm by GloMax® (Promega, Madison, WI, USA) for calculating cell viability. The wavelength of 600 nm was used as reference.

### Immunocytochemistry

Cells were fixed with 4% paraformaldehyde for 10 min. And cells were permeabilized with 0.1% tritonX/PBS for 30 min at RT. The 2% goat serum was used for blocking reaction for 60 min. Samples were incubated with primary antibody (rabbit anti-SOD1 antibody, 1:150, Enzo Life Sciences, Inc, Farmingdale, NY, USA) at 4 °C overnight. And we incubated cells with second antibody (goat anti-mouse antibody Alexia 555, (Thermo Fisher Scientific)) at RT for 30 min. Samples were observed by using confocal imaging system (Zeiss LSM 700).

### ROS production

To detect mutant SOD1 induced ROS production, we used a Red mitochondrial superoxide indicator, MitoSOX® Red (Thermo Fisher Scientific). N2a cells were prepared in uncoated glass-bottomed microwells. After transfection of each plasmid, MitoSOX® Red was added to the cell culture to a final concentration of 5 μM to the cells and incubate for 30 min at 37 °C following the manufacturer’s instructions (Thermo Fisher Scientific). Fluorescent microscopy images were acquired using a confocal fluorescence microscope (LSM700, Carl Zeiss) and the images were analyzed using ZEN software (Carl Zeiss).

### ESR analysis

The measurement condition for ESR (JES-FA 200, JEOL) was applied as following: center field 330 mT; sweep width, 1.5 × 10 mT; sweep time, 4 min; field modulation width, 2 × 0.1 mT; amplitude, 5.0 × 100; time constant, 0.3 s; microwave power, 4.0 mW. The •OH scavenging activity was calculated from the integration values of the ESR spectra corrected with a Mn^2+^ marker. The •OH was generated by the Fenton reaction. Sample solution (50 μL), 72 mM DMPO (50 μL), 2 mM H_2_O_2_ (50 μL), and 0.2 mM FeSO_4_ (50 μL) were mixed and transferred to an ESR spectrometry cell. Exactly 1 min after the addition of FeSO_4_, ESR spectrum of DMPO-OH spin adducts was recorded.

### Statistical analysis

Data are given as the mean ± standard error of the mean (SEM). The significance of differences was determined by Student’s t test or an analysis of variance (ANOVA). Further statistical analysis for post hoc comparisons was performed using the Bonferroni/Dunn test (StatView, Abacus, Baltimore, MD, USA). A *p*-value of less than 0.05 was considered to be statistically significant.

## Electronic supplementary material

Supplement
