# Supplementary material for: The effects of Brazilian green propolis that contains flavonols against mutant copper-zinc superoxide dismutase-mediated toxicity
Source: Sci Rep. 2017 Jun 6;7:2882. doi: 10.1038/s41598-017-03115-y (PMC5460160; doi:10.1038/s41598-017-03115-y)

**(Supplementary Information)**

**The effects of Brazilian green propolis that contains flavonols against mutant copper-zinc superoxide dismutase-mediated toxicity**

Tomoyuki Ueda, Masatoshi Inden, Katsuhiro Shirai, Shin-ichiro Sekine, Yuji Masaki, Hisaka Kurita, Kenji Ichihara, Takashi Inuzuka & Isao Hozumi

Corresponding author: Isao Hozumi, M.D., Ph.D.

E-mail: hozumi@gifu-pu.ac.jp

**Supplemental Fig S1** LDH assay. N2a cells expressing mCheery-SOD1^G85R^ were treated with kaempferol (from 0.3 µM to 10 µM) or kaempferide (from 5 µM to 50 µM). The cell viability was measured by LDH assay. * *p* < 0.05; ** *p* < 0.01; *** *p* < 0.001. Scale bar: 20 µm.


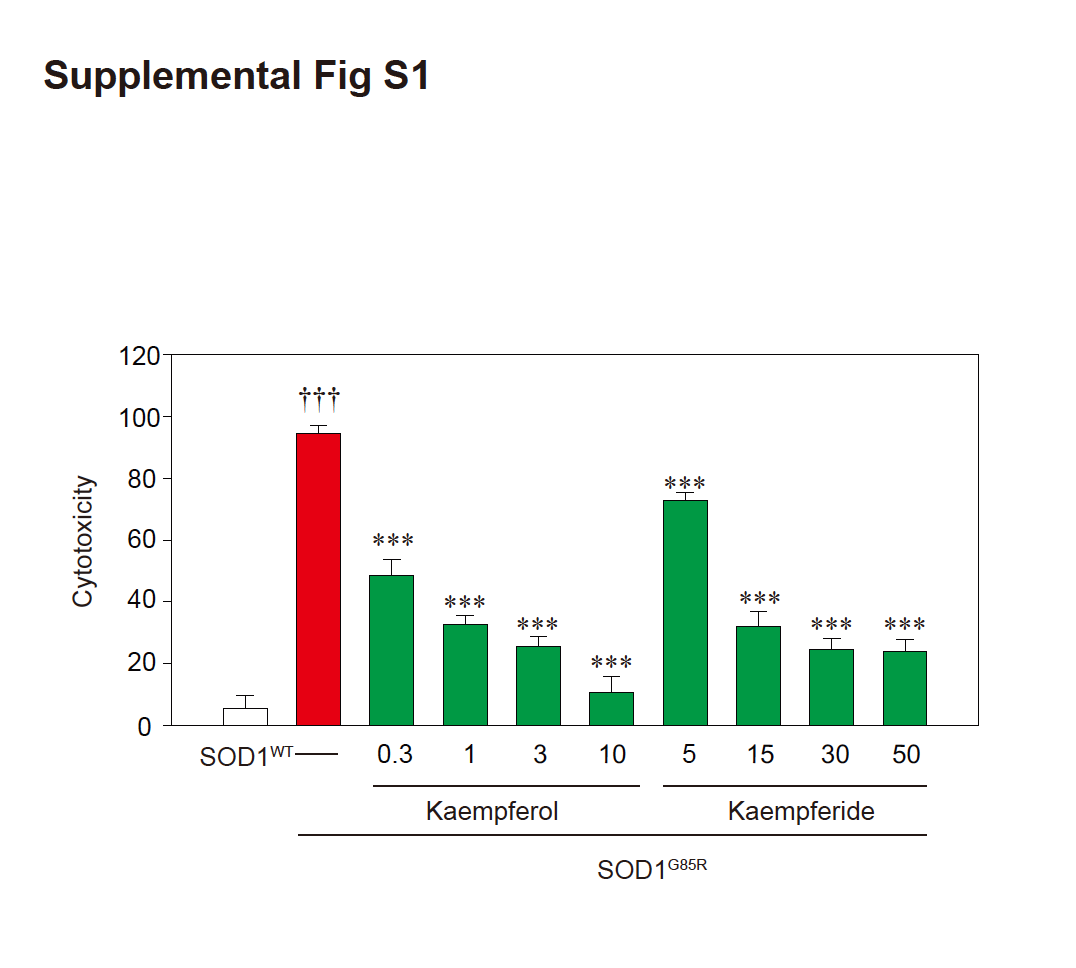

Supplement: Supplementary file 1 — Supplement [file 41598_2017_3115_MOESM1_ESM.doc]
